# Section on Ophthalmology

**Published:** 1896-10

**Authors:** Geo. H. Noble

**Affiliations:** Secretary, 186 S. Pryor St., Atlanta, Ga.


### SECTION ON OPHTHALMOLOGY,

### COLLEGE OF PHYSICIANS OF PHILADELPHIA.

A Stated Meeting of the Section on Ophthalmology was held in the Lower Hall of the College of Physicians on the 17th of March, 1896, Dr. Wm. F. Norris, Chairman, presiding. Present: Drs. Friebis, Hansell, Harlan, Norris, Oliver, Randall, de Schweinitz, Shaffner, and Zimmerman, Fellows of the College; and Beaudoux, Bromley, Capp, Green, Krauss, Leopold, Lovelace, McGuigan, Mellor, Moorhead, Murdock, Palmer, Posey, Rogers, Schwenk, Shoemaker, Sulzer, Sweet, Tait, Taylor, Veasey, and Ziegler as guests.

Dr. George C. Harlan exhibited a case of traumatic enophthalmus in a five-year-old boy, who five months previously was wounded by the horn of a bull. The right cheek and temple and the lower eyelid were lacerated, and the inferior margin of the orbit was chipped. There was also complete ptosis. At the time of examination, the tendo oculi was found to have been torn away and the lower lid was dragged downward and outward by the action of the orbicularis and the contraction of the cicatrix.

The surgeon who attended the patient at the time of the accident, reported that there was considerable orbital cellulitis, with abundant discharge of pus from between the lids, but there never was any exophthalmus. He thought that the cellulitis was confined to the lower part of the orbit. At present the eyeball is retracted and has the appearance of being very much smaller than its fellow. The cornea is situated five millimeters behind the plane of that of the other eye. There is scarcely more ptosis than would result from the depression and loss of support of the lid. When the patient looks directly forward, the palpebral fissure is five or six millimeters wide. He insists that he sees well with the eye. Though the movements of the eyeball are much restricted, no diplopia can be detected. There is complete inability to look upward beyond the horizontal line, either directly or to the right or left. Horizontal movements are normal and the downward excursion is much exaggerated. Dr. George Friebis spoke of his case of traumatic enophthalmus seen nine years ago, in which there was sufficient recovery to manifest but little difference between the two eyes; there being nothing left except a slight doubling of objects when looked at below the horizontal line. In his case there was no incarceration of the extraocular muscles.

Dr. Francis M. Perkins showed a case of monocular retinal detachment with high myopia.

Dr. Charles A. Oliver gave the clinical history of a case of ciliary staphyloma and excavation of the optic disc following traumatic cataract in a four-year-old boy. The clinical picture of this case of complicated secondary glaucoma was so complete, having been studied from almost what may be termed its very incipiency to the final result, and the varying symptoms evolved from time to time were so at variance with what one would expect in such cases, that it offered itself as a most interesting and a most instructive study of this type of disease.

Unlike similar cases of sudden obstruction to proper lymph-stream circulation, there remained from the very first, as shown by the fields of vision, and, as afterward proven ophthalmoscopically, an element that may possibly complicate many more cases of the traumatic type of this disease than is at present imagined, and that is retinal detachment. Again, the condition of the vitreous and its peculiarity of opacities, taken in connection with the history of the case, would go far to show that there was a hemorrhage into that humor which most probably might have been recognized ophthalmoscopically had the patient been seen a week earlier. These, with a few, though certain evidences of a low grade iridocyclitis, made the case still more atypical.

On the other hand, the progressive diminution of the field of vision; the gradual distention of the globe, and the localized tissue-bulgings in the upper ciliary regions; the deep and characteristic cupping of the nerve-head; the reapproximation of the remaining areas of retinal detachment; and the late fixedly increased intraocular tension, all show the certainty of degeneration even in a young and yielding eyeball, when such tissues are subjected to a persisting increased intraocular pressure.

As answer to the vexed question of therapy in such cases, the author will leave this for another and more extended communication, reserving the present brief though detailed account, of the clinical history as an interesting and useful exposition of a grouping of symptoms, which have been carefully studied, and can be thus employed to illustrate the results of two conflicting contemporaneous conditions produced by traumatism: localized inflammatory reaction and obstruction of lymph-stream circulation.

Dr. George E. de Schweinitz presented a further note on an unusual form of macular lesion following iritis. The patient, a fifty-year-old woman, recovered with a nearly normal sharpness of vision, but with some vitreous opacities from a violent attack of serous iritis. The eye remained comfortable for eight months, when she appeared with a positive scotoma and the ability to see to count fingers only when situated in the periphery of the visual field. In addition to the positive scotoma, which the patient described as appearing "like a dinner-plate with a green edge," there was a small absolute scotoma about the horizontal level. Ophthalmoscopic examination revealed an oval, reddish area, giving the impression of a disintegrating hemorrhage, and containing in the center several white dots situated exactly in the center of the macular region. Dr. de Schweinitz referred to the unusually distinct macular ring which seemed to indicate that there must be some thickening in the periphery of the hemorrhagic area.

Dr. Oliver exhibited a water-color sketch of a case of unusual submacular hemorrhage forming a part of some very curious lymph extravasations in the retina without any vitreous disturbances, found in the left eye of a healthy sixty-five-year-old woman upon whom he had successfully removed a black cataract by simple extraction some two months previously, the operation being perfectly smooth and the appearance of the interior portion of the eye normal in every respect. The sketch was made for him by Miss Margaretta Washington, of this city.

Dr. de Schweinitz described the clinical history of a patient suffering from convergent strabismus of the left eye and a very high myopia 16 D. Ophthalmoscopically, the following lesions were present: A small posterior polar cataract, numerous fine vitreous opacities, and a horizontally oval optic disc, of a greenish-gray color. The nerve-head was imbedded in the center of a huge mass of opaque fibers, which followed the course of the principal vessels almost to the periphery of the eye-ground, and in all directions, but less markedly downward and to the nasal side. A small patch in the macular region was not covered by the opaque fibers, but was disturbed by superficial choroidal changes. There was almost complete loss of nasal field and of the entire center of the visual field, with exception of a small area to the nasal side of fixation, about ten degrees in diameter, within which the white test object was dimly seen. Colors were correctly appreciated when held in the temporal field. The case was illustrated by a water-color drawing made by Miss Washington.

In the discussion, Dr. B. Alexander Randall showed a card-specimen of a case of retained nerve-sheaths in a case that had been sent to him for supposed intracranial disturbances. In this case there was an isolated patch situated in the macular region. Dr. Oliver exhibited the drawing of a case in which the medullation began at the edge of the disc and divided into two comet-like processes extending along the lines of the larger retinal vessels, this case having been seen through the courtesy of Drs. Goodman and Ziegler, at the Wills Eye Hospital. He also spoke of a drawing that was made for him by Dr. Randall, which was one of the most extensive of annular types that he had ever seen. The case occurred in a nine-year-old, highly myopic boy, who never had any subjective symptoms of the condition.

Dr. James Thorington, by invitation, exhibited an asbestos cover-chimney with disc-attachment for ophthalmoscope purposes. The original form with the disc-attachment he had made two years previously. The present arrangement showed that five changes could be made in the disc. (1) The one centimeter opening fulfilled all the purposes of the original chimney. (2) The two centimeter opening permitted greater freedom of movement on the part of the observer, without moving the light. (3) The three centimeter opening may be used as a source of light for the concave skiascope, or for the ophthalmoscope, otoscope, etc. (4) A round section of cobalt blue glass for the chromatic aberration test of ametropia had been added, as likewise: (5) The perforated disc, with perforations and spaces, each 1.45 millimeter, to test for astigmatism at one meter's distance. The author stated that he had a new form of contrivance in the course of preparation, which will have a simple shutter with different changes in it, to work up and down in front of the opening in the asbestos chimney by means of cog-wheels. He will also employ a horizontal slip one-eighth of an inch wide to exercise the oblique muscles as suggested to him by Dr. Savage, of Nashville, Tenn. Dr. Charles Shaffner strongly recommended the asbestos form of chimney, as it radiated but little or no heat and was always sufficiently cool to handle without burning the fingers. It had been his intention to present one that he had been using for some time, but as he considered that the present form and the one recently brought forward by Dr. M. W. Zimmerman were much better, he had refrained from so doing.

Dr. Thorington showed a new form of perimetric lenses, which received its name from the fact that their optical center corresponds to the point of fixation in the fields of vision. The reasons given for the recommendation of the lens were, that it gives to the eye that form of lens which is consistent with a normal form of the visual field; it removes the edge of the lens to a sufficient distance that the edge cannot be seen to any great degree while the eye is fixed straight ahead; and that bifocal segments can be made much larger. He stated that the increase in weight need rarely exceed the ordinary form of twenty-five to thirty grains; the large size does not attract much attention; and the cost will remain the same as in the ordinary styles used. Upon account of necessary great weight and thickness, he believed that this form of lens cannot be used for cases of aphakia and high myopia, but showed that as this class of cases constitutes much less than one-half of all refraction cases (37 per cent.), the lens will be accepted in the majority of instances.

Dr. Oliver exhibited and demonstrated a series of microscopic specimens showing the various forms of eyes seen in fish, reptiles, birds, quardrupeds, and man. He showed the marked differences in the conditions of the dioptric media; the varying shapes of the eyeball; the relative positions of the eye in the head of the animal; the adaptions for near and for far-focusing; the arrangements for increase of the interior illumination; the positions and peculiarities of the nerve-structure; and the relationship existing intra-cranially between the two organs, in the aquatic, the terrestrial, and the aerial forms of animal life.

The Section then went into executive session. Upon motion, adjourned.

### SECTION ON OBSTETRICS AND DISEASES OF WOMEN.

Milo B. Ward, Chairman, Topeka, Kan.; George H. Noble, Secretary, Atlanta, Ga.; Executive Committee, Jos. Eastman, Indianapolis, Ind.; F. H. Martin, Chicago, Ill.; J. T. Johnston, Washington, D. C.

As an effort has been made to keep a mailing list of the members of the American Medical Association interested in the Section on Obstetrics and Diseases of Women, please be kind enough to publish a notice to the effect that all members desiring to participate in the proceedings or to attend the meetings of this Section should send their names and addresses to the undersigned, as no communication concerning papers, program, etc., can be had with members not upon the proposed list.

Most respectfully,

GEO. H. NOBLE, Secretary,

186 S. Pryor St., Atlanta, Ga.

Other medical journals please copy.

---

The program of the proceedings of the Sixth Annual Meeting of the American Electro-Therapeutic Association to be held in Allston Hall, the Studio Building, Clarendon street, near St. James avenue, Boston, Mass., Tuesday, Wednesday, and Thursday, September 29, 30, and October 1, 1896, has reached us, and holds out promise of an interesting meeting. Papers are to read and discussed, which deal with electricity in its various forms as applicable to medicine and to the surgery of different part of the body.

---

### COSMETICS OF CASTRATION.

The esthetically inclined editor of the *Medical Record* suggested some time ago that the unsightly void left by orcheotomy should be filled by a celluloid testicle for cosmetic reasons. This suggestion (from a recent report of a discussion before the Chicago Medico-Legal Society) would seem to have fallen on fruitful soil. Discussing the question of castration of criminals, Dr. Gertrude G. Wellington remarked that a man castrated for disease wished something to take the place of the organ removed that would give him the semblance of manhood. The only thing that could be found were some balls of celluloid, but they each contained a little bell. She would advise that in habitual criminals and sexual perverts after castration, these celluloid balls with their jingling bells be inserted so that as the man went about among women of the world the bells would proclaim him incapacitated.—*Medical Standard.*

